# A color spectrographic phonocardiography (CSP) applied to the detection and characterization of heart murmurs: preliminary results

**DOI:** 10.1186/1475-925X-10-42

**Published:** 2011-05-31

**Authors:** Reza Ramezani Sarbandi, John D Doyle, Mahdi Navidbakhsh, Kamran Hassani, Hassan Torabiyan

**Affiliations:** 1 Department of Biomechanics, Science and Research Branch, Islamic Azad University, Tehran, Iran; 2 Department of General Anaesthesiology, Cleveland Clinic Lerner College of Medicine of Case Western Reserve University, Ohio, USA; 3 Mechanical Engineering Department of Iran University of Science & Technology, Tehran, Iran

## Abstract

**Background:**

Although cardiac auscultation remains important to detect abnormal sounds and murmurs indicative of cardiac pathology, the application of electronic methods remains seldom used in everyday clinical practice. In this report we provide preliminary data showing how the phonocardiogram can be analyzed using color spectrographic techniques and discuss how such information may be of future value for noninvasive cardiac monitoring.

**Methods:**

We digitally recorded the phonocardiogram using a high-speed USB interface and the program *Gold Wave *http://www.goldwave.com in 55 infants and adults with cardiac structural disease as well as from normal individuals and individuals with innocent murmurs. Color spectrographic analysis of the signal was performed using *Spectrogram *(Version 16) as a well as custom MATLAB code.

**Results:**

Our preliminary data is presented as a series of seven cases.

**Conclusions:**

We expect the application of spectrographic techniques to phonocardiography to grow substantially as ongoing research demonstrates its utility in various clinical settings. Our evaluation of a simple, low-cost phonocardiographic recording and analysis system to assist in determining the characteristic features of heart murmurs shows promise in helping distinguish innocent systolic murmurs from pathological murmurs in children and is expected to useful in other clinical settings as well.

## Background

During the cardiac cycle, the normal heart produces repeatable physiological sounds. However, under some pathologic conditions, such as valvular disease or ventricular septal defects, the presence of turbulent blood flow leads to the production of additional sounds, called murmurs, which are random rather than deterministic in nature. Although the stethoscope has been used for almost two centuries, it is still frequently difficult to determine whether a heart murmur is abnormal or innocent murmur. Furthermore, detecting structural abnormities of the heart such as mitral stenosis or insufficiency remains an important clinical problem. Although many such abnormities can be detected by careful auscultation, generally only experienced cardiologists are able to detect important but subtle auscultatory findings with reliability [1-6]. Although echocardiography is frequently used to identify and characterize such abnormities, it is an expensive technology that is generally not suitable for either mass-screening or continuous monitoring. In addition, its operation requires a considerable knowledge and skill. Similar problems exist with cardiac catheterization methods.

The present study seeks to apply a relatively new technique, known herein as Color Spectrographic Phonocardiography (CSP), to develop a noninvasive instrument to assist in the detection and characterization of heart murmurs. The study builds on the work of others in the field of classical and digital phonocardiography. In particular, techniques such as time-frequency analysis of acoustic emissions, neural network methods, acoustic spectral averaging, and wavelet techniques have all been are used for the analysis of heart sounds and murmurs [7-18].

## Methods

We collected the heart sounds at the pediatric clinic of Modares Hospital in Tehran between 2009 to 2010. The study was approved by the ethics committee of the hospital and the study protocol conforms to the ethical guidelines of the 1975 Declaration of Helsinki as reflected in *a priori *approval by the institution's human research committee. Data were collected from a total of 55 patients, including 5 normal cases, with an age range of 1 month to 14 years. Written informed consent was obtained from the patients for publication of the case reports and accompanying images. A copy of the written consent is available for review by the Editor-in-Chief of this journal. The patients and the normal ones had a history of heart murmur, which included 20 with a ventricular septal defect (VSD), 7 with an atrial septal defect (ASD), 4 with Tetralogy of Fallot (TOF), 10 with aortic stenosis (AS), 5 with pulmonary stenosis (PS), and 4 having mitral regurgitation (MR). In all cases, the diagnosis was confirmed by echocardiography. The summary of medical history and diagnostic findings for some samples can be seen in Table 1.

**Table 1:** Recorded heart sounds for some of the patients

Patient Study Number	Patient age	Patient weight	Cardiac disease	Max frequency of Murmurs	Grade of Intensity	Remark
**1**	6	23	Normal	100 Hz	-	

**2**	8 months	-	VSD	700 Hz	4/6	

**3**	3	12	ASD	300 Hz	3/6	

**4**	6	21	AS	280 Hz	2/6	

**5**	14	31	PS	610 Hz	4/6	

**6**	11	22	TOF	400 Hz	6/6	

**7**	10	28	MR	290 Hz	2/6	Dextrocardia

**Figure 1:**
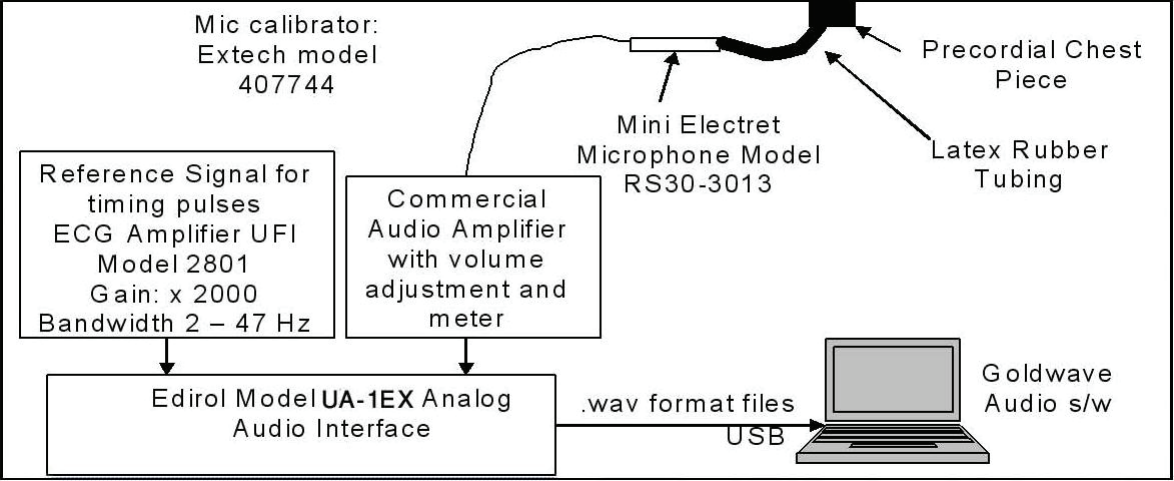
**The schematic of the system**. The schematic of the system which has been used for collection of the preliminary data.

The data were recorded using a laptop computer-based phonocardiographic recording system developed at the Science and Research Branch of Islamic Azad University. Figure 1 shows the system used. A miniature electret microphone, connected to a precordial chest piece, was connected to a commercial audio amplifier whose output was then digitized at 44 KHz with 16 bits resolution. A similar arrangement was also used to record the electrocardiogram (ECG), which was recorded to assist in the identification of the start of each cardiac cycle. (Because the ECG was digitized using a sound card that was high-pass filtered at around 20 Hz, this signal was somewhat different than those recorded under full-bandwidth conditions. Despite this, however, the QRS complex of the ECG still could be used as a marker of the beginning of each cardiac cycle.) This setup was placed on a mobile cart for easy recording in cardiology clinics and elsewhere. Table 2 shows the equipment list which were used for recording the data.

**Table 2:** Equipment list which was used in this project

Row	Equipment	Remark
**1**	Precordial Stethoscope Chest Pieces	

**2**	Latex Rubber Tubing	

**3**	Mini Electret	Nexxtech Ultra-Miniature Tie-Clip

	Microphone	Microphone Model 3303013

**4**	Sound Level Calibrator, 94dB	Model 407744

**5**	Commercial Audio Amplifier with volume adjustment and meter	

**6**	ECG Bio Amplifer UFI	Model 2122i

**7**	USB Audio Interface	EDIROL Model UA-1EX

Microphone calibration was performed using a Sound Level Calibrator, 94dB Model 407744 which produces a sinusoidal wave in 1 KHz and 94 dB SPL intensity. Comparing the recorded sound to the calibration recordings, it was then possible to obtain the absolute intensity measurements.

**Figure 2:**
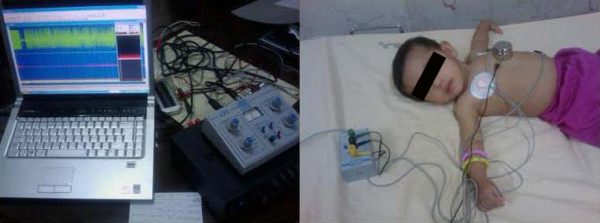
**A photo of the recording system (left) and one of the patients during a recording session (right)**. The photo of the system which was used for recording the data(left) and one of the patients during a recording session(right).

In order to achieve high-quality recordings, the recording environment was kept completely silent, with the patients lying in the supine position. Each recording was divided into five sections of 3 minutes each. Care was taken to ensure that borborygmi sounds (from stomach and intestines) and other artifacts were not present. The Figure 2 shows a schematic of the recording system (left) and one of the cases during recording (right).

All sounds were recorded using the Goldwave software (version 5.55), which includes tools for recording, playing, filtering, and analyzing sounds. Using this software, we also deleted any electrical utility frequency (50 Hz) from the ECG recordings. Next, we wrote code in the MATLAB software programming environment, based on the FFT function, which enabled us to display the phonocardiographic graphs in color spectrographic form. (Additional file 1: Appendix). It should be emphasized that the phonocardiographic graphs are presented in the time domain but the spectrograms are plots in both frequency and time. The color spectrograms let us calculate the required frequency in terms of the time using the colors intensity.

## Results

### Case 1

**Figure 3:**
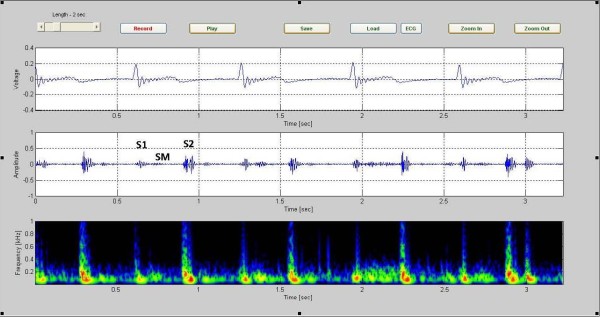
**sample phonocardiographic (middle) and electrocardiographic (top) recording obtained from a healthy six year old girl**. Sample phonocardiographic (middle) and electrocardiographic(top) recording obtained from a healthy six year old girl with an innocent murmur. The corresponding color spectrogram is shown in the bottom. (Case1).

The Figure 3 shows sample phonocardiographic (middle) and electrocardiographic (top) recording obtained from a healthy six year old girl with an innocent murmur. The corresponding color spectrogram is shown in the bottom. The recording is of 5 cardiac cycles covering an elapsed time of 3.35 seconds. Note that the frequency of the murmur is largely under 200 Hz. Furthermore, S1 and S2 can be readily identified in the phonocardiogram signal.

### Case 2

**Figure 4:**
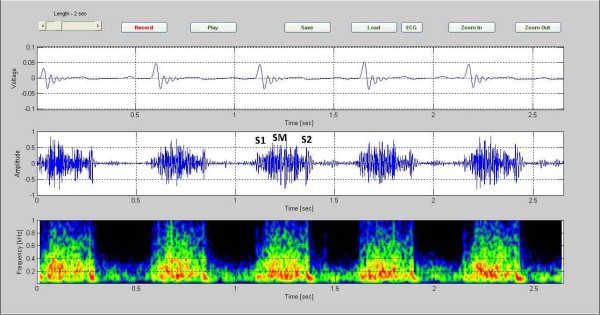
**VSD in a 8-month-old patient, (CASE 2)**. VSD in a 8-month-old patient. Five cardiac cycles are shown. The spectrographic graph, bottom of the figure, indicates that the murmur has frequency components extending to 700 Hz (Case 2).

The Figure 4 presents the data for a case of VSD in an 8 month old girl. Five cardiac cycles are shown. The spectrographic graph, bottom of the figure, indicates that the murmur has frequency components extending to 700 Hz. Noting to the graph, we found out the position of the murmur is between S1 and S2.

### Case 3

**Figure 5:**
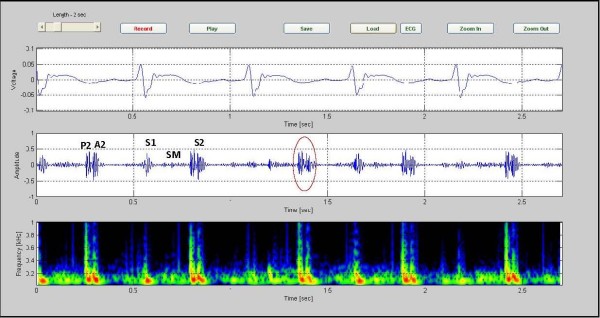
**ASD in a 3-year-old patient, (CASE 3)**. ASD in a 3-year-old patient. The murmur has frequency components that extend to around 300 Hz. The second heart sound is clearly split in both the phonocardiogram and in the corresponding spectrogram (CASE3).

Five cardiac cycles of data for a 3 year old girl with an ASD are shown in Figure 5. The murmur has frequency components that extend to around 300 Hz. The second heart sound is clearly split in both the phonocardiogram and in the corresponding spectrogram.

### Case 4

**Figure 6:**
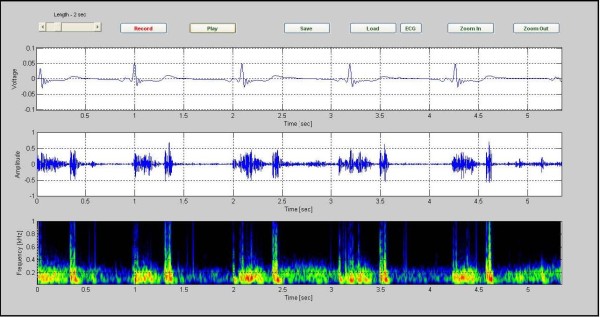
**Aortic stenosis in a 6-year-old patient, (CASE 4)**. Aortic stenosis in a 6-year-old patient. The recording covers 5 heart cycles and takes 5.40 seconds. The murmur is between S1 and S2 and extends to about 400 Hz (CASE 4).

The Figure 6 presents data for a 6 year old boy with AS. The recording covers 5 heart cycles and takes 5.40 seconds. The murmur is between S1 and S2 and extends to about 400 Hz.

### Case 5

**Figure 7:**
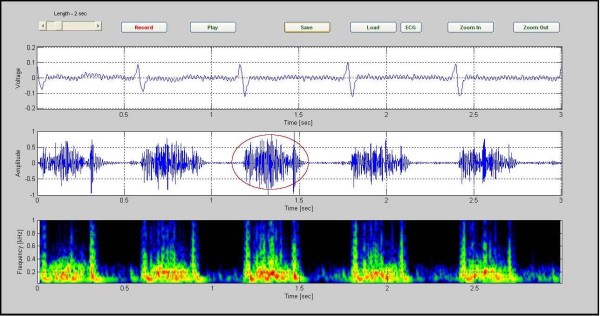
**Pulmonary stenosis in a 14-year-old patient,(CASE 5)**. Pulmonary stenosis in a 14-year-old patient. This diamond-shaped systolic murmur has components to over 600 Hz. P2 could be heard (CASE 5).

Data for a 14 year old boy with PS are presented in Figure 7. This diamond-shaped systolic murmur has components to over 600 Hz. P2 could be heard.

### Case 6

**Figure 8:**
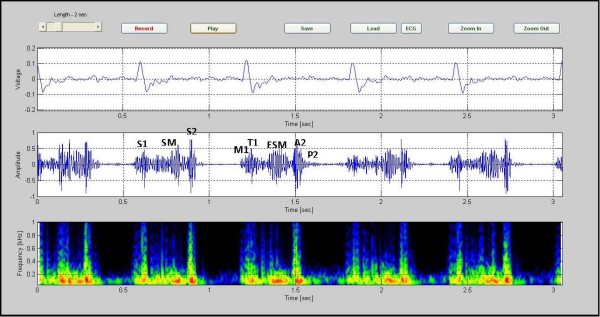
**Tetralogy of Fallot in a 11-year-old patient, (CASE 6)**. Tetralogy of Fallot in a 11-year-old patient which shows a systolic murmur with frequency components extending to around 400 Hz (CASE 6).

Data for an 11 year old girl with TOF are presented in Figure 8, which shows a systolic murmur with frequency components extending to around 4 00 Hz.

### Case 7

**Figure 9:**
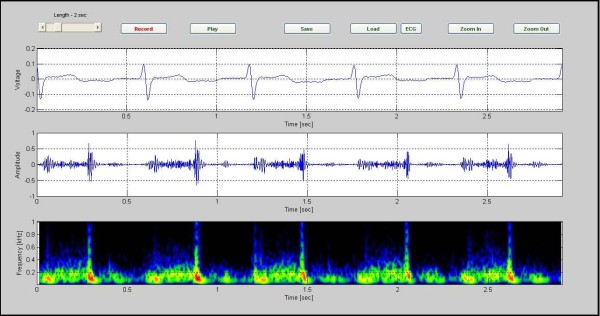
**Mitral regurgitation in a 10-year-old patient, (CASE 7)**. Mitral regurgitation in a 10-year-old patient. A systolic murmur with components extending to 400 Hz is evident (CASE 7).

The Figure 9 is from a 10 year old boy with MR. A systolic murmur with components extending to 400 Hz is evident.

## Discussion

Compared to pathological systolic murmurs, innocent systolic murmurs were found to have a lower peak frequency, below 200 Hz, have a shorter duration, and always faded in intensity before the second heart sound. In addition, the first and second heart sounds were readily discernible in innocent pediatric murmurs.

In all of the graphs, S1 has lower frequency than S2. We believe that the following explanation explains this finding. The elastic modulus (E) of the atria and ventricles are much less than for the arterial walls. It is known from vibration theory that a vibrating mechanical system with small elastic modulus oscillates with lower frequency than a system with higher elastic modulus. Therefore, a smaller elastic modulus results in a lower frequency of oscillation [19]. Also, since the blood volume in the larger arteries is normally less than the blood in the ventricles this results in less inertia for the oscillating volume. Consequently, the frequency of the second heart sound (S2) tends to be higher than for the first heart sound (S1).

We found out each of the heart sounds take more than 0.1 second, S1:0.14 s and S2: 0.11s. Both of these sounds are low-frequency sounds but S1 more so than S2. Furthermore, the S2 duration was found to be less than that for S1, presumably in part because the attenuation of S2, from the arterial walls, is more rapid than for S1, from the ventricular walls.

Investigating the spectrogram of the VSD patient in Figure 4, we note that this was a holosystolic murmur. Note also that the shape of the VSD murmur in this case is rather similar to that in Additional file 2: Table S1. The murmur which is heard in ASD patients is mostly mid systolic in character and is caused from an increase in blood leaving the right ventricle. The increase of the load presented to the right ventricle makes the duration of the systole longer. This is associated with a splitting of S2, as noted in the color spectrogram shown in Figure 5 (see red circles). Aortic stenosis (AS) is associated with a harsh mid systolic murmur in aorta area. In the AS patient shown in Figure 6, we observed that the murmur starts after S1 and reaches to maximum in the middle, 350~400 Hz frequency, and shrinks to a minimum before S2. The shape of murmur here is also similar to that in Table S1. In Pulmonary Stenosis (PS), the blood is not able to easily enter into the pulmonary artery. This condition is characterized by a harsh systolic murmur, Figure 7, which is comparable to the shape shown in Additional. file 2: Table S1. In the case of TOF(VSD, right ventricular hypertrophy, PS, and overriding aorta)since VSD and PS are both have systolic murmurs, the result is a systolic murmur resulting from two sources, Figure 8. Finally, in the MR case, Figure 9, note the presence of a holosystolic murmur with an apparent early diastolic murmur (likely due to rapid antegrade flow through the mitral orifice).

Our eventual aim is to collect a sufficient number of cases to allow us to separately investigate the time-domain and spectrographic characteristics of each form of cardiac disease, so as to establish what characteristics each condition has in common that are not present in a collection of normal recordings. This would first require a careful examination of the two sets of data (pathologic and normal) to find candidate parameters that would help distinguish the two groups. This would likely be followed by formal pattern recognition methods to identify which parameters or set of parameters best separate the recording into "normal" and "abnormal" groups. In addition, we are exploring the possibility of developing "digital subtraction phonocardiography" methods that we hope will be able to separate any murmurs from underlying deterministic heart sounds. Our hope here is to develop an easily-implemented and inexpensive non-invasive approach to distinguish the murmurs from the underlying normal heart sounds by processing the acquired phonocardiogram and electrocardiogram of a patient. This approach will be developed and evaluated first by applying advanced signal processing techniques on mathematical simulations of signals containing both deterministic heart sounds and random (as in stochastic) murmurs. The information obtained from this preliminary analysis will be evaluated on healthy volunteers to determine the clinical factors that may affect the implementation of the new approach. Our hope is that the new technique will be evaluated in a cohort of patients with pathological murmurs to determine its clinical potential through a comparison of the results with those from classical auscultation and echocardiography on these patients.

Finally, some limitations of the present study should be emphasized. First, we expect that inexperienced clinical examiners need some guidance in the form of rules about how to read the spectrograms. Such rules have not yet been well researched. Another limitation is that the source of the continuous low frequency energy found in many recordings is not yet known; one possible cause we are considering is poor skin contact of the chest piece. Finally, studies on a large number of patients with homogeneous cardiac defects like VSDs will be necessary to make this technology more useful.

## Conclusions

Recently, Noponen et al. [7] studied the phono-spectrographic features of heart murmurs to investigate how features obtained from a phono-spectrogram can be used to distinguish innocent murmurs from pathological murmurs in children. In another study, Kudriavtsev et al. [8] developed an approach based on spectrograms to present an intensity/frequency image of heart sound properties across time, which they called heart energy signature spectrogram. Our study extends this cardiac spectrographic experience for a number of cardiac conditions. In summary, we present a relatively new method, called color spectrographic phonocardiography, which is relatively simple, non invasive, and low in cost. It can be learned easily and interpretation of the results is potentially easier than the use of time-domain data alone. The method can also be used for prolonged continuous monitoring.

## List of Abbreviations

AS: aortic stenosis; ASP: atrial septal defect; CSP: Color Spectrographic Phonocardiography; ECG: electrocardiogram; Hz: Hertz; PCG: phonocardiogram; MR: mitral regurgitation; USB: Universal Serial Bus; TOF: Tetralogy of Fallot; VSD: ventricular septal defect.

## Competing interests

The authors declare that they have no competing interests.

## Authors' contributions

RRS and HT conducted the data sampling and wrote the MATLAB code required for converting the murmurs into signals. JDD and MN supervised the research and JDD revised the manuscript and offered the research proposal. KH wrote the manuscript and coordinated the research. All authors have read and approved the final manuscript.

## Supplementary Material

Additional file 1 **Appendix**. Click here for file

Additional file 2 **Table S1**. The shape of different murmurs [Redrawn from reference 20]. Click here for file
