# Supplementary material for: A color spectrographic phonocardiography (CSP) applied to the detection and characterization of heart murmurs: preliminary results
Source: Biomed Eng Online. 2011 May 31;10:42. doi: 10.1186/1475-925X-10-42 (PMC3126734; doi:10.1186/1475-925X-10-42)
Supplement: Additional file 1 — Appendix. [file 1475-925X-10-42-S1.PDF]

Appendix

MATLAB software code

Appendix

MATLAB software code

```
% Type "record" in the matlab command window to
% run the program.

function record(op)

global H_RECORD F_RECORD AXIS_RECORD ai data z_data R_fs R_samp_len


global ZOOM_HISTORY

if nargin == 0 % if no input argument, draw the GUI
   op = 0;
end

%spec = 'wideband';
spec = 'narrowband';
wideband_time = 4e-3;
narrowband_time = 25e-3;
%window_width = 200;    % window width for FFT
%step_size    = 100;  % window movement
fft_pts      = 4096; % # of points in the FFT

switch op

case 0 % Draw figure

   clear global data  % erase any previously recorded data

   width = 950;
   height = 700;

   F_RECORD = figure('Position',[25 50 width height],...
      'NumberTitle','off',...
      'Color',[.8 .8 .8],...
      'Name','Record');

   H_RECORD(1) = uicontrol('Style','pushbutton',... % record button
      'Units','normalized',...
      'Position',[190/width (height-75)/height 75/width
25/height],...
      'ForegroundColor',[1 0 0],...
      'FontWeight','bold',...
      'String','Play',...
      'String','Record',...
      'Visible','on',...
      'CallBack','record(1)');
```

```
   H_RECORD(2) = uicontrol('Style','text',...
      'Units','normalized', ...
      'Position',[190/width (height-45)/height 75/width
25/height],...
      'BackgroundColor',[.8 .8 .8],...
      'String','      ');

   H_RECORD(3) = uicontrol('Style','pushbutton',... % play button
      'Units','Normalized', ...
      'Position',[300/width (height-75)/height 75/width
25/height],...
      'ForegroundColor',[.2 .4 .2],...
      'FontWeight','bold',...
      'String','Play',...
      'Enable','off',...
      'CallBack','record(2)');

   H_RECORD(4) = uicontrol('Style','Slider',... % user selects time
length

      'Units','normalized', ...
      'Position',[60/width (height-70)/height 100/width
20/height],...
      'Min',1,'Max',6,'Value',2,...
      'SliderStep',[1/5-0.000001 1/5],...
      'Callback','record(3)');

   H_RECORD(5) = uicontrol('Style','text',...  % diplays time length
      'Units','normalized', ...
      'Position',[50/width (height-45)/height 120/width
20/height],...
      'BackgroundColor',[.8 .8 .8],...
      'String','Length - 2 sec');

   H_RECORD(6) = uicontrol('Style','pushbutton',... % Zoom in on data
      'Units','normalized', ...
      'Position',[(width-240)/width (height-75)/height 75/width
25/height],...
      'ForegroundColor',[.2 .4 .2],...
      'FontWeight','bold',...
      'String','Zoom In',...
      'Enable','off',...
      'CallBack','record(4)');

   H_RECORD(7) = uicontrol('Style','pushbutton',... % Zoom out
      'Units','normalized', ...
      'Position',[(width-135)/width (height-75)/height 75/width
25/height],...
      'ForegroundColor',[.2 .4 .2],...
      'FontWeight','bold',...
      'String','Zoom Out',...
      'Enable','off',...
      'CallBack','record(5)');
```

```
   H_RECORD(8) = uicontrol('Style','pushbutton',... % Load data from
wav file
      'Units','normalized', ...
      'Position',[(width-395)/width (height-75)/height 75/width
25/height],...
      'ForegroundColor',[.2 .4 .2],...
      'FontWeight','bold',...
      'String','Load',...
      'Enable','on',...
      'CallBack','record(6)');

   H_RECORD(9) = uicontrol('Style','pushbutton',... % Ecg data from
wav file
      'Units','normalized', ...
      'Position',[(width-315)/width (height-75)/height 35/width
25/height],...
      'ForegroundColor',[.2 .4 .2],...
      'FontWeight','bold',...
      'String','ECG',...
      'Enable','on',...
      'CallBack','record(7)');

   H_RECORD(10) = uicontrol('Style','pushbutton',... % Save data to
wav file
      'Units','normalized', ...

      'Position',[(width-500)/width (height-75)/height 75/width
25/height],...
      'ForegroundColor',[.2 .4 .2],...
      'FontWeight','bold',...
      'String','Save',...
      'Enable','off',...
      'CallBack','record(8)');




case 1 % record button

    % Set sampling and length info
    %R_fs = 11025;
    R_fs = 16000;
    R_samp_len = get(H_RECORD(4),'Value');

    % get handles for sound input and output
    ai = init_sound(R_fs,R_samp_len);
    R_fs = get(ai, 'SampleRate'); % in case actual rate doesn't match
desired

    % gets an array named data from the microphone
    nogo=0;
```

```
   while not (nogo)
      set(H_RECORD(1),'String','Recording');
      set(H_RECORD(2),'String','Speak now...');
      start(ai);
      try
         data = getdata(ai);
         nogo=1;
      catch
         disp('10 seconds elapsed... try again!');
         stop(ai);
      end
   end
    delete(ai);
   set(H_RECORD(1),'String','Record');
   set(H_RECORD(2),'String','          ');

   % Make "Play", "Zoom In/Out", and "Save" buttons available
   set(H_RECORD(3),'enable','on');
   set(H_RECORD(6),'enable','on');
   set(H_RECORD(7),'enable','on');
    set(H_RECORD(10),'enable','on');

    % normalize sound data to 99% of max
   data = 0.99*data/max(abs(data));
   z_data = data;
    % displays the time graph of the voice signal
   AXIS_RECORD(1) = timedata(F_RECORD,data,R_fs,0.06,0.38,0.88,0.20);
   xlims = get(AXIS_RECORD(1),'XLim');
   ylims = get(AXIS_RECORD(1),'YLim');
   ZOOM_HISTORY = [];
   ZOOM_HISTORY = push(ZOOM_HISTORY,[xlims ylims]);

    % Sampling rate dependent window width

    if strcmp(spec,'narrowband')
        window_width = round(R_fs*narrowband_time);
        step_size = round(window_width/8);
    elseif strcmp(spec,'wideband')
        window_width = round(R_fs*wideband_time);
        step_size = round(window_width/2);
    end

   % calculates the spectrum of the voice signal
    X = specgram(data,fft_pts,1,hamming(window_width),window_width-
step_size);
    X = abs(X);
   % displays the spectrum of the voice signal
    AXIS_RECORD(2) =
spectdata(F_RECORD,X,R_fs,step_size,window_width,0.06,0.10,0.88,0.20)
;
    axis([xlims get(gca,'YLim')])
```

```
case 2  % Play recording

   % sends an array named z_data to the speakers/headphones
   if length(z_data) ~= 0
      sound(z_data,R_fs)
    end

case 3  % Display time length text

   % Allow the user to set the time length of sample
   num = get(H_RECORD(4),'Value');
   set(H_RECORD(5),'String',['Length - ' num2str(num) ' sec']);

case 4

   % Zoom in on the data

   axes(AXIS_RECORD(1))
   axes(AXIS_RECORD(3))

   % Allow the user to draw a rectangle on the area
   % they would like to zoom in on
   RECT = getrect;

   xmin = RECT(1);
   xmax = RECT(1) + RECT(3);
   ymin = RECT(2);
   ymax = RECT(2) + RECT(4);

   % Set maximum zoom limits to the data edges
   xaxis_limits = get(AXIS_RECORD(1),'XLim');
   yaxis_limits = get(AXIS_RECORD(1),'YLim');
   xaxis_limits = get(AXIS_RECORD(3),'XLim');
   yaxis_limits = get(AXIS_RECORD(3),'YLim');
   if xmin < xaxis_limits(1)
      xmin = xaxis_limits(1);
   end

   if xmax > xaxis_limits(2)
      xmax = xaxis_limits(2);
   end
```

```
    if ymin < yaxis_limits(1)
       ymin = yaxis_limits(1);
    end

    if ymax > yaxis_limits(2)
       ymax = yaxis_limits(2);
    end

    % if the choosen zoom range is acceptable...
    if ~((ymin > ymax) | (xmin > xmax))

       % zoom in on the time data
       axis([xmin xmax ymin ymax]);
       % define the last limits
       last_axis_limits = [xaxis_limits yaxis_limits];
       ZOOM_HISTORY = push(ZOOM_HISTORY,last_axis_limits);


       % define the zoomed in data (for playback purposes)
       imin = round(xmin*R_fs)+1;
       imax = round(xmax*R_fs)+1;
       z_data = data(imin:imax);

    end

 case 5

    zoom_level = length(ZOOM_HISTORY);

    if zoom_level > 1
       [ZOOM_HISTORY,axis_limits] = pop(ZOOM_HISTORY);
    else
       axis_limits = ZOOM_HISTORY{1};
    end

    % zoom out to the full time data
    axes(AXIS_RECORD(1))
    axis(axis_limits);

    % zoom out to the full frequency data
    axes(AXIS_RECORD(2))
    ymin = 0;
    ymax = R_fs/2000;
    axis([axis_limits(1:2) ymin ymax]);

    % define the zoomed in data (for playback purposes)
    imin = round(axis_limits(1)*R_fs)+1;
    imax = round(axis_limits(2)*R_fs)+1;
    z_data = data(imin:imax);
    %z_data = data;

 case 6 % Load button

    [filename, pathname] = uigetfile('*.wav','Select Data File');

    if filename ~= 0
```

```
        cd(pathname);

       %  Get data and sampling rate
       [data,R_fs] = wavread([pathname filename]);
       if min(size(data))>1
          error('Can''t load stereo data')
       end
     % Make "Play", "Zoom In/Out", and "Save" buttons available
        set(H_RECORD(3),'enable','on');
        set(H_RECORD(6),'enable','on');
        set(H_RECORD(7),'enable','on');
        set(H_RECORD(10),'enable','off');

        z_data = data;
         % displays the time graph of the voice signal
        AXIS_RECORD(1) =
timedata(F_RECORD,data,R_fs,0.06,0.38,0.88,0.20);
        xlims = get(AXIS_RECORD(1),'XLim');
        ylims = get(AXIS_RECORD(1),'YLim');
        ylabel('Amplitude')
        ZOOM_HISTORY = [];
        ZOOM_HISTORY = push(ZOOM_HISTORY,[xlims ylims]);
        grid on

         % Sampling rate dependent window width
         if strcmp(spec,'narrowband')
             window_width = round(R_fs*narrowband_time);
             step_size = round(window_width/8);
         elseif strcmp(spec,'wideband')
             window_width = round(R_fs*wideband_time);
             step_size = round(window_width/2);
         end

        % calculates the spectrum of the voice signal
         X =
specgram(data,fft_pts,1,hamming(window_width),window_width-
step_size);
         X = abs(X);
        % displays the spectrum of the voice signal
         AXIS_RECORD(2) =
spectdata(F_RECORD,X,R_fs,step_size,window_width,0.06,0.10,0.88,0.20)
;
         axis([xlims get(gca,'YLim')])
         grid on

   end

 case 7 % ECG button

   [filename, pathname] = uigetfile('*.wav','Select Data File');

   if filename ~= 0

        cd(pathname);

       %  Get data and sampling rate
       [data,R_fs] = wavread([pathname filename]);
       if min(size(data))>1
          error('Can''t load stereo data')
```

```
      end
    % Make "Play", "Zoom In/Out", and "Save" buttons available
       set(H_RECORD(3),'enable','on');
       set(H_RECORD(6),'enable','on');
       set(H_RECORD(7),'enable','on');
       set(H_RECORD(10),'enable','on');

       z_data = data;
        % displays the time graph of the voice signal
       AXIS_RECORD(3) =
timedata(F_RECORD,data,R_fs,0.06,0.65,0.88,0.20);
       xlims = get(AXIS_RECORD(3),'XLim');
       ylims = get(AXIS_RECORD(3),'YLim');
       ylabel('Voltage')
       ZOOM_HISTORY = [];
       ZOOM_HISTORY = push(ZOOM_HISTORY,[xlims ylims]);
       grid on

        % Sampling rate dependent window width
        if strcmp(spec,'narrowband')
            window_width = round(R_fs*narrowband_time);
            step_size = round(window_width/8);
        elseif strcmp(spec,'wideband')
            window_width = round(R_fs*wideband_time);
            step_size = round(window_width/2);
        end

   end

 case 9 % Save waveform

    [filename, pathname] = uiputfile('*.wav', 'Save Data to Wave
File');
    if filename ~= 0
        wavwrite(z_data,R_fs,[pathname filename])
    end
end


%----------------------------------------------------------------
% SUBFUNCTION
function H = timedata(Fig,x,fs,left,bottom,width,height)
% This function plots time data at location specified by user
% Left, bottom, width, height are relative locations less than 1

figure(Fig);

samp_len = length(x)/fs;
delta_t = 1/fs;
t = 0:delta_t:(samp_len-delta_t);

% display the signal
H = subplot('position',[left bottom width height]);
plot(t,x), xlabel('Time [sec]'), ylabel('Amplitude')
axis([0 t(length(x)-1) -1 1 ]);


%----------------------------------------------------------------
% SUBFUNCTION
```

```
function H = spectdata(Fig,x,fs,step,ww,left,bottom,width,height)
% This function plots spectral data at location specified by user
% Left, bottom, width, height are relative locations less than 1

figure(Fig);

% frequency axis vector
y_len = size(x,1); % num of rows
f = [0:y_len-1]*fs/y_len/2;
f = f/1000; % kHz scale

% time axis vector
x_len = size(x,2); % num of columns
%t = [0.5:1:x_len+0.5]*step/fs;
%t = [(ww-1)/2-0.5:step:step*x_len+(ww-1)/2-1.5]/fs;
t = [(ww-1)/2:step:(x_len-1)*step+(ww-1)/2]/fs;

% display the signal
H = subplot('position',[left bottom width height]);
log_data = -log10(x+0.0001);
imagesc(t,f,log_data), xlabel('Time [sec]'), ylabel('Frequency
[kHz]')
set(gca,'YDir','normal')



%-------------------------------------------------------------------
% SUBFUNCTION
function ai = init_sound(fs,samp_len)
% Function 'init_sound' initializes microphone input for voice
% 'fs' is the sampling rate, 'samp_len' is the time to record
%   in seconds.

v = ver;
name = {v.Name};
ind = find(strcmp(name,'MATLAB'));
if isempty(ind)
    ind = find(strcmp(name,'MATLAB Toolbox'));
end

v_num = str2num(v(ind).Version);

ai = analoginput('winsound');
addchannel(ai, 1);
if (v_num == 6.1) | (v_num == 6.5)
    set(ai, 'StandardSampleRates', 'Off');
end
set(ai, 'SampleRate', fs);
actual_fs = get(ai, 'SampleRate');
set(ai, 'TriggerType', 'software');
set(ai, 'TriggerRepeat', 0);
set(ai, 'TriggerCondition', 'Rising');
set(ai, 'TriggerConditionValue', 0.01);
set(ai, 'TriggerChannel', ai.Channel(1));
set(ai, 'TriggerDelay', -0.1);
set(ai, 'TriggerDelayUnits', 'seconds');
set(ai, 'SamplesPerTrigger', actual_fs*samp_len+1);
set(ai, 'TimeOut', 10);
```

```matlab
%-----------------------------------------------------------------
% SUBFUNCTION
function s = push(s,new_item)
% Function 'push' adds 'new_item' to stack 's'
if ~isempty(s)
    height = length(s);
   s{height+1} = new_item;
else
   s{1} = new_item;
end

%-----------------------------------------------------------------
% SUBFUNCTION
function [s,x] = pop(s)
% Function 'pop' removes item from top of stack 's'
height = length(s);
x = s{height};
s = s(1:height-1);

%-----------------------------------------------------------------
% SUBFUNCTION
function [rptgenml_creport1] = buildreport
%BUILDREPORT
% Create RptgenML.CReport
rptgenml_creport1 = RptgenML.CReport(...
  'Format','rtf97',...
  'Stylesheet','!print-NoOptions',...
  'RptFileName','.rpt');
% setedit(rptgenml_creport1);
```
