# Supplementary material for: A color spectrographic phonocardiography (CSP) applied to the detection and characterization of heart murmurs: preliminary results
Source: Biomed Eng Online. 2011 May 31;10:42. doi: 10.1186/1475-925X-10-42 (PMC3126734; doi:10.1186/1475-925X-10-42)
Supplement: Additional file 2 — Table S1. The shape of different murmurs [Redrawn from reference 20]. [file 1475-925X-10-42-S2.DOCX]

| **Type of Murmur** | **Murmur Shape** |
| --- | --- |
| **Atrial Septal Defect (ASD)** | Expiration  Inspiration  **P2**  **T1**  **M1**  **M1**  **T1**  **A2**  **P2**  **A2** |
| **Aortic Stenosis (AS)** | S1  S2 |
| **Ventricular Septal Defect(VSD)** | S1  S2 |
| **Pulmonary Stenosis(PS)** | S1  S2 |
| **Tetralogy of Fallot(TOF)** | M1  T1  A2  P2 |
| **Mitral Regurgitation(MR)** | M1  S3  P2  A2  T1 |
